# Ovarian Cancer Immunotherapy: Turning up the Heat

**DOI:** 10.3390/ijms20122927

**Published:** 2019-06-15

**Authors:** Eleonora Ghisoni, Martina Imbimbo, Stefan Zimmermann, Giorgio Valabrega

**Affiliations:** 1 Candiolo Cancer Institute FPO/IRCCS, 10060 Candiolo (TO), and University of Torino, 10100 Turin, Italy; eleonora.ghisoni@ircc.it; 2 Department of Oncology, Lausanne University Hospital, and Ludwig Institute for Cancer Research, University of Lausanne, 1005 Lausanne, Switzerland; martina.imbimbo@chuv.ch (M.I.); stefan.zimmermann@chuv.ch (S.Z.)

**Keywords:** ovarian cancer, immunotherapy, adoptive cell therapy, tumor infiltrating-lymphocytes, tumor microenvironment

## Abstract

Epithelial ovarian cancer (EOC) is the leading cause of death among gynecological malignancies. Despite surgery and chemotherapy, 5-years survival rates have improved only modestly over the past few decades remaining at 45% for advanced stages. Therefore, novel therapies are urgently needed. The presence of tumor-infiltrating lymphocytes (TILs) in OC tumor microenvironment (TME) has already proved to be correlated with overall survival (OS), while immune evasion mechanisms are associated with poor prognosis. Although these data indicate that immunotherapy has a strong rationale in OC, single agent immune-checkpoints inhibitors (ICIs) have shown only modest results in this malignancy. In this review, we will discuss immune-targeting combination therapies and adoptive cell therapy (ACT), highlighting the challenges represented by these strategies, which aim at disrupting the stroma-tumor barrier to boost immune system against ovarian cancer.

## 1. Introduction

Epithelial ovarian cancer (EOC) is the leading cause of death among women with gynecological malignancies with 22,530 estimated new cases and 13,980 deaths in 2019 in the USA [1]. Surgery and chemotherapy, based on carboplatin and paclitaxel, have been long established as the cornerstone for the primary management of EOC [2].

However, despite multimodal treatment and the transforming advance represented by the introduction of agents targeting poly (ADP-ribose) polymerase (PARP) [3], prognosis is still poor for advanced stages and survival rates have improved only modestly over the past few decades [4].

EOC has traditionally been considered as scarcely immunogenic. However, several findings contradict this statement, such as spontaneous tumor regressions [5,6], evidence of mechanisms of immune evasion and occasional durable responses to immune checkpoints-inhibitors (ICIs) [7]. 

Notably, BRCA1/2-mutated high-grade serous ovarian cancers (HGSOCs) exhibit a higher mutational load and a unique mutational signature with a significantly increased number of tumor-infiltrating lymphocytes (TILs), as well as elevated expression of programmed cell death (PD-1) or its ligand (PD-L1) in tumor-associated immune cells compared to homologous-recombination (HR)-proficient tumors [8,9]. Furthermore, patients with T-cell-rich tumors experience longer progression-free and overall survival [10], while immune evasion mechanisms are associated with poor survival [11,12,13,14,15]. All these evidences taken together suggest that EOC patients could potentially benefit from immunotherapy. 

Despite the encouraging results in melanoma, non-small cell lung cancer (NSCLC), kidney and urothelial cancers [16,17], the use of single-agent antibodies inhibiting the cytotoxic T lymphocyte-associated protein 4 (CTLA-4) or PD-1 or PD-L1 axis yielded only modest results in EOC with median response rates of 10–15%, and a control of disease observed in less than half of the patients [18,19,20,21].

Interestingly, the combination of the anti-PD1 nivolumab and anti-CTLA4 ipilimumab showed promising results in platinum resistant EOC at the six months-interim analyses with an overall response rate (ORR) of 34% (doubling the results of nivolumab monotherapy). However, data are still immature [22].

As a consequence, no immunotherapeutic agent has obtained regulatory approval for EOC thus far.

Many strategies to overcome resistance to ICIs are currently under investigation and the tumor–immune system interaction is now considered a key element guiding the research toward more personalized approaches.

According to the status of TILs infiltration, tumors could be histologically categorized as “inflamed/hot tumors” or “non-inflamed/cold”. The first ones are characterized by the presence in the tumor bed of a high density of CD8^+^ T cells [23,24], whose functionality can be impaired by immunosuppressive networks. Such patients could benefit from therapies acting on T cell checkpoint involved in immune-tolerance. 

On the contrary, cold tumors are characterized by the absence of T cells in tumor beds and at tumor edges and are generally affected by a failure in T cell priming reflecting the need of strategies that could deliver autologous/allogenic effector cells into the cancer. 

A third phenotype, defined as “immune-excluded”, is characterized by the modification of tumor microenvironment (TME) and the presence of inhibitory cells that retain CD8 T cells from entering the tumor islets, even if they are present in the stroma. Such patients could benefit from strategies whose aim is to increase infiltrations of tumors by immune effector cells such as T cell trafficking modulators, epigenetic modulators, TME remodeling molecules, radiation therapy [25].

**Figure 1:**
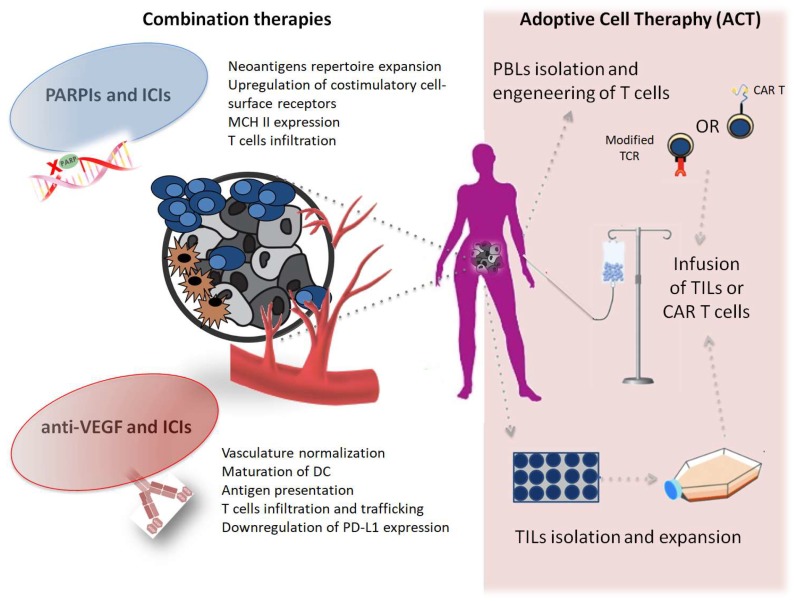
Representative scheme of combination therapies approaches and adoptive cell therapy (ACT) in OC.

In this review, we focus on the strategies and challenges represented by immune-targeting combinations in the most advanced stages of development in EOC. We also describe challenges and advances in adoptive cell therapy (ACT) which, despite the limited data available in EOC so far, represent a unique opportunity to enhance immune-response “heating the fire” against OC (Figure 1).

PARP inhibitors (PARPIs) and immune-checkpoints inhibithors (ICIs) promote the release of pro-inflammatory signals and the expression of co-stimulatory molecules, expand the neoantigens repertoire and indeed increase the visibility of tumor cells to T cells. Anti-vascular endothelial grow factor (anti-VEGF) agents normalize the vascular structure of the tumor microenviroment (TME) regulating T cells infiltration and trafficking and contribuiting to dendritic cells (DCs) maturation and downregulation of the PD-L1 pathway expression. ACT (a): Tumor-infiltrating lymphocites (TILs) are isolated from surgically resected tumor samples, then expanded in vitro and reinfused into the lymphodepleted patient; (b): T cells from patient peripheral blood are isolated and expanded in culture and genetically modified to express either a T cell receptor (TCR) or a chimeric antigen receptor (CAR) that confers the ability to specifically recognize and destroy tumor-cells when re-infused into the the lymphodepleted patient.

## 2. Combination Therapies

### 2.1. Check-Point Inhibitors and Anti-VEGF Therapy

Ovarian TME consists of blood and lymphatic vessels and of different types of stromal cells embedded in the omental extracellular matrix. These cells can act as immune modulators and comprise myeloid-derived suppressor cells (MDSCs), tumor-associated macrophages (TAMs), adipocytes, CAFs (cancer activated fibroblasts) and resident and infiltrating immune cells, including regulatory T cells (T_reg_).

Tumor vessels are abnormal, both structurally and functionally, and favor an immunosuppressive environment characterized by hypoxia, low pH and high interstitial fluid pressure due to dysfunctional lymphatic drainage. 

An abnormal TME promotes the activation and expansion of immunosuppressive Treg cells and the recruitment of TAMs, the expansion of abnormal endothelial cells (ECs), and the suppression of dendritic cell (DC) maturation, which in turn results in impaired antigen presentation and activation of tumor-specific cytotoxic lymphocytes. Importantly, the PD-1/PD-L1 pathway is often upregulated both on tumor cells and on tumor-infiltrating CTLs, marking them as dysfunctional or ‘exhausted’ and limiting their cytotoxic potential [26]. Furthermore, the vascular endothelial grow factor (VEGF) can promote the expansion of MDSCs and enhance their immunosuppressive function in the TME. [27,28].

Thus, there is a strong rationale in combining antiangiogenic therapies and ICIs and, in fact, many phase 3 trials are currently ongoing. Antiangiogenics might partially increase the effectiveness of immunotherapy through the normalization of the abnormal tumor vasculature and contrasting the direct inhibitory effect on immune and endothelial cells, increasing the infiltration of immune effector cells into tumors and converting the intrinsically immunosuppressive TME to an immune-supportive one [29].

In 2017, Lee et al. [30] conducted a phase 1 basket trial to evaluate the antitumor effect of the PD-1 inhibitor durvalumab in combination with endothelial growth factor receptor 1–3 inhibitor cediranib or PARP inhibitor olaparib in women with solid tumors. A total of nine patients with EOC were treated in the durvalumab plus cediranib arm. Five patients had a partial response (PR) with an overall response rate (ORR) of about 55%, which is higher compared with that recorded for PD-L1 pathway inhibition (10–20% ORR) [18] or cediranib treatment alone (23% ORR) [31]. However, treatment with durvalumab plus cediranib was associated with a higher frequency of adverse events (AEs). Continuous cediranib was not tolerated due to hypertension and diarrhea (seven patients suffered from grade 2–4 AEs and one patient experienced grade 3 pulmonary hypertension and eventually died from pulmonary embolism). 

More recently, Liu et al. presented at ESMO 2018 [32] the encouraging preliminary results of the phase II trial evaluating the combination of anti-PD1 nivolumab and bevacizumab in 38 patients affected by EOC, including 18 with platinum-resistant disease. The combinatorial treatment shows an ORR of 28.9% (40% and 16.7% in the platinum-sensitive and in the platinum-resistant cohort respectively) with a median PFS of 8.1 months and no unexpected AEs.

Thus, bevacizumab seems to be a safe and tolerable anti-VEGF drug to be associated to checkpoint blockade to enhance immuno-response and currently three phase 3 trials are ongoing both in front-line and recurrence settings (Table 1).

### 2.2. Check-Point Inhibitors and PARP-Inhibitors

The landscape of poly (ADP-ribose) polymerase (PARP) inhibitors (PARPIs) is rapidly evolving and represents a cornerstone in the natural history of EOC. Since 2014 the Food and Drug Administration (FDA) and the European Medicine Agency (EMA) have approved three PARPIs for the treatment of EOC [33,34,35,36,37,38]. 

PARP proteins catalyze the polymerization of poly (ADP-ribose) on proteins. This reversible post-translational modification of proteins, also called PARylation, is implicated in many cellular mechanisms, notably DNA repair. PARP detects single-strand breaks (SSBs) and, through its PARylation activity, recruits proteins that mediate DNA repair. Furthermore, PARP-1 shows wide immune effects: i) Regulation of DCs maturation from monocytes; ii) reduction of CD86 and CD83 and of IL-12 and IL-10 expression; iii) modulation of the activation of nuclear factor of activated T cell (NFAT) which is essential in T cell function. [39,40,41]

As stated before, EOC with BRCA1/2 mutation has been identified as an ideal candidate for immunotherapy and PARPIs combination. In HGSOC, tumors harboring HR-deficient/BRCA1/2 mutations demonstrated a higher neoantigen load and increased numbers of CD3+ and CD8+ TILs [42]. Elevated levels of PD-1 and PD-L1 expression on TILs were also observed compared with that in HR-proficient tumors, which indicated that BRCA1/2-mutated HGSOCs may be more sensitive to PD-1/PD-L1 inhibitors compared with HR-proficient ones. 

A phase I study to evaluate the safety and efficacy of the combination of PARP inhibitor and anti-CTLA-4 antibody was conducted in women with BRCA mutation-associated recurrent OC [43]. No dose-limiting toxicities were identified and grade 1/2 toxicities were consistent with prior studies that used immune checkpoint inhibitors. More recently, two studies exploring different combinations were presented at ASCO 2018. The first one by Konstantinopoulos et al. was a phase 1/2 study evaluating niraparib and pembrolizumab [44]. Interestingly, ORR was 23% without any significant difference in biomarkers selected populations (25% in BRCA-mutated and 24% in BRCA wild-type respectively). The second one, already published [45], combined olaparib and durvalumab in platinum-sensitive relapsed BRCA mutated EOC. Overall, 23 of 32 patients had objective responses with olaparib plus durvalumab, including six (19%) complete responses. Subgroup analysis showed a consistency of response in patients who had received up to three prior lines of therapy. The combination achieved a 12-week disease-control rate (DCR) of 81%. Furthermore, six patients had complete responses (CR) and 17 others had PRs resulting in an ORR of 72%. An additional three patients had SD, bringing the DCR to 81%. The combination was well tolerated, with a low incidence of grade 3 or higher AEs or all-grade immune-related adverse events (irAE). The most common irAEs (all grades) were hypothyroidism in five (15%) patients and rash in four (12%). Finally, the study presented by Lee et al. (durvalumab and olaparib in recurrent EOC) at ESMO 2018 did not meet the primary endpoint with an overall DCR of 37% and only 5/35 (14%) PR [46].

The above results suggest that the combinations of PARPIs and PD-1/PD-L1 blockade seem to enhance the antitumor effect without significantly increasing toxicity and support their further development in EOC independently from BRCA mutational status.

Ongoing phase III trials exploring ICIs and PARPIs combinations are listed in Table 2.

### 2.3. Adoptive Immunotherapies

Adoptive immunotherapy is emerging as an active treatment in cancers that are less responsive or refractory to ICIs. This approach is based on the infusion of autologous or allogenic immune effectors that destroy tumor cells.

Currently one of the most promising approach in solid cancers is represented by ACT, in which tumor-specific cytotoxic T cells, either isolated from the tumor or in the peripheral blood by leukapheresis, are expanded in vivo and then infused after lymphodepleting chemotherapy. 

EOC is characterized by a low/intermediate mutational burden [47,48]. However, several experiences have shown that these tumors can harbor missense somatic mutations that can serve as neoantigens and elicit tumor specific T cells that could be exploited for ACT.

In a prospective series, authors showed that in five out of seven cases, EOC metastases from immunotherapy naïve patients are infiltrated by mutation reactive T cells (mainly against mutant p53), thus at frequencies that are encouraging toward future use in adoptive therapies [49].

In another work, Bobisse et al. reported the identification of neo-epitope specific CD8^+^ T cells in ~90% of evaluated patients, with a notably higher functional avidity in TILs compared to circulating T lymphocytes and different TCR repertoires [50].

Westergaard et al., successfully established and expanded TILs from 34 tumors specimen of OC and demonstrated the recognition of autologous tumor cell in >50% of the patients. Furthermore, antigen specific TILs were isolated and further expanded in vivo [51].

Taken together these preclinical data support the development of ACT in EOC. 

First evidences of possible activity of ACT in EOC derives from first line, non-randomized phase 1 study in which TILs were administered in 13 patients with no evidence of disease after surgical resection and cisplatin-based chemotherapy. Eleven patients with the same characteristics were treated with chemotherapy only and served as control arm. The 3-year overall survival rates were shown to be higher in the TILs group (100% compared to 67.5%, respectively) [52].

Recently, results from a pilot study in platinum resistant OC with lympho-depleting chemotherapy followed by the infusion of unselected TILS and IL2 were published. The therapy was feasible and with no unexpected toxicities related to high dose chemotherapy or the administration of IL2. There were signs of antitumor reactivity in TILs infusion products but clinical results are still not satisfactory since patients achieved only SD as best response for three to five months. Authors specify that a high percentage of infused TILs expressed exhaustion markers with tumor tissue expressing inhibitory immune checkpoint ligands. These findings open the road toward the possibility of enhancing ACT combining it to ICIs [53].

Other possible strategies could be applied to enhance efficacy of adoptive therapies.

First, although considered as immunosuppressive for several decades, it is now known that chemotherapy could improve immunotherapy efficacy, acting on several factors, such as T cells trafficking, neo-antigen presentations and TME. Indeed several studies indicate that also in OC neoadjuvant chemotherapy (NACT) enhance CD8 + TILs infiltration and activity and may reduce T_regs_ [54,55,56]. In the most recent Lo et al. show that NACT increased pre-existing TIL responses (in already TILs-infiltrated tumor) but with scant effect on cold tumors and on immunosuppressing cells [57]. Thus, this evidence suggests that the optimal timing for TILs collection should be further investigated, possibly being different in inflamed- and non-inflamed tumors to maximize TILs vitality and activity when infused.

Second, activity of ACT could be enhanced with the use of antigen selected TILs, that has proven feasible in OC [51] or by genetically engineering T cells to express either an exogenous tumor antigen-specific T cell receptor (TCR) or a chimeric antigen receptor (CAR).

In the first case the TCR is engineered to have a higher affinity for both intracellular and extracellular targets but T-cell activity is still dependent on the antigen presentation and expression of MHC that can be impaired in tumor tissue. Additionally, TCR activity is also MHC haplotype restricted. Furthermore, T cells can be isolated from the patient’s peripheral blood and engineered with a specific receptor to target a known TAA on the patient’s tumor. This allows the generation of a highly specific T cell that can be obtained from peripheral blood, avoiding tumor extraction and expansion of TILS and also theoretically limiting toxicities.

In contrast, CARs are engineered so that they can recognize an antigen in a MHC independent fashion through an antigen specific extracellular antibody single chain variable fragment (scFv) but is thus limited to surface antigens. 

Several clinical trials using engineered T cells to target antigens in OC, such as NY-ESO-1, HER2, FR-alpha, MSLN, MUC16, and p53 are currently recruiting in solid cancers (Table 3).

Despite good in vitro and in vivo activity of anti-folate receptor (Fr) alpha CAR T cells, the first phase 1 trials in metastatic OC patients failed to show any sign of activity [58]. Possible reasons, according to authors, are scarce T cell trafficking and persistence of cell in the tumor. New generation CAR-T comprehensive of costimulatory signaling domain have shown greater persistence and efficacy in vivo and in vitro models of OC [59].

Targeting FR-alpha with cell-based therapies, drugs or vaccines are generally well tolerated and seemed promising showing early signs of responses with Bi-specific T-cell engaging (BiTE) antibodies [60]. Furthermore, since it is expressed by the vast majority of HGSOC at much higher levels than healthy ovarian tissue, it represents an ideal candidate for immunotherapy.

Mesothelin is another promising target that is expressed in about 70% of epithelial OC [61]. Preliminary results of second generation anti mesothelin CAR T in OC are encouraging: The therapy was feasible and safe, the CAR T-meso cells engrafted and expanded in the blood of all subjects. There was evidence of trafficking to tumor sites resulting in the clearance of pleural effusion in one patient and in stabilization of disease in all six patients [62].

Exploiting the innate immune system is the base of adoptive-non-antigen-based strategies, consisting in the infusion of natural killer (NK) or cytokine-induced killer cells (CIK) cells. Use of these cells could have several advantages, such as the independence from MHC and from prior sensitization to the antigen. These therapies have proven to be feasible but research is still at an early stage. With regards to NK adoptive therapy, a first phase 2 trial with haploidentical allogenic NK cells showed scarce persistence of NK in the donor, with no relevant clinical activity [63]. In a phase I trial two patients with ovarian cancer were treated with allogenic haploidentical NK cells. Best responses were SD and PD [64], while in a case report patient achieved a PR with biomarker response after six treatments. 

These studies suggest that allogeneic NK cell therapy is feasible although further efforts that will generate novel strategies to increase in vivo NK cell persistence and expansion after adoptive transfer are needed.

CIK cells are a group of immune effector cells with cytotoxic activity that can be rapidly proliferated in vitro from PBMC after incubation with different cytokines (IFN Y, IL2, IL1 and anti CD3). The main effectors of CIK cells are the NK-like T lymphocytes (CD3+ CD56+) that have potentially enhanced and broader antitumor activity compared to TILS and do not depend from TCR and MHC activity but still can elicit both MHC-restricted and MHC-unrestricted anti-tumor citotoxicity [65]. Such cells showed to be very active against ovarian cancer in vitro and in vivo [66] and also showed enhanced activity and better tolerability compared to LAK cells (PBMC incubated with IL2), that demonstrated limited clinical efficacy with high rates of peritoneal fibrosis when given intraperitoneally [67,68,69].

There are signals that CIKs could be useful in the adjuvant setting, but further evidences are needed [70].

## 3. Patients’ Selection

As discussed, inhibition of checkpoint PD-1/PDL1 and CTLA 4 axis produces low response rates in unselected ovarian cancer patients, as the majority of clinical trials so far have been recruiting patients without distinction between inflamed- and excluded-tumors.

Currently, biomarkers like PD-L1 expression, TMB and TILs infiltration have not yet proven to be useful for patient selection in OC. While expression of PD-L1 on tumor cells is a predictive biomarker of response to ICIs in other cancer types (i.e., NSCLC and urothelial cancers), its expression in EOC is not very frequent (10–33%) [71,72] suggesting that not all tumors rely on this pathway for immune evasion and its prognostic role in EOC is still controverted. Some data suggest that high expression of PD-L1 correlates with worse prognosis [73,74], while in other series a higher expression correlates with better PFS [75,76,77].

High levels of CD8^+^ TILs are known to be associated with better prognosis [10], as well as the presence of other immune cells such as CD4^+^ T cells [78], memory B cells [79] and plasma cells [80]. However, their presence has not proven to be predictive biomarkers of response to ICIs. Zhang et al. showed that epithelial CD8+ TILs correlates with neoantigens selection and high rates of HLA expression loss, thus suggesting a strong immune-editing in the TME. The neoantigen depletion and the impaired presentation together with the tumor heterogeneity and the clonal evolutions of tumor in different metastatic sites could influence immunosurveillance and the response to ICIs of the so-called “inflamed tumors” [81].

Mutational status of EOC could predict immunogenicity. As already mentioned, tumor with homologous-recombination deficiency (HRD) have a higher predicted neo-antigen load and higher TILs infiltration. However clinical data shows that response to ICI monotherapies is rare in BRCA mutated patients as well, indicating that neoantigen load is not a good selection strategy, but also opening the road for combination therapies with PARPIs.

Furthermore, a recent analysis on the Keynote-100 showed no statistically significant differences in HRD status among responders and non-responders and the absence of association between BRCA status and responses. Interestingly, TMB and T cell-inflamed gene expression profile (GEP) were independently predictive of response and demonstrated low correlation, suggesting that they capture distinct features of neo-antigenicity and T cell activation [82,83].

## 4. Conclusions

New and more effective treatment modalities of immunotherapy are being investigated in EOC based on tumor biology, its relationship with TME and the immune-suppressive networks that make this cancer difficult to tackle with single agent ICIs.

Specific antigens such as NY-ESO-1, FR-alpha, MSLN, MUC16, and p53 are attractive immunotherapy targets, due to their frequent expression in OC and are currently under investigation in different modalities such as vaccines [84], oncolytic viral platforms, combination therapies with ICIs or chemotherapies [84,85,86] and as TAAs in adoptive cellular immunotherapy [87]. 

TME plays a crucial role in the immune-suppression and immune-tolerance that can impair the presence and the activity of TILs, such as the presence of T_regs_, TAMs, MDSC, exosomes, adipocytes and CAFs. All these TME’s components should be taken into account when designing novel therapeutic strategies, in particular for the immune-excluded/“cold tumors”. These may include strategies to prime T cell responses, including vaccines and ACT directed to neo-antigens presented by tumor cells; inducers of immunogenic cell death in strategies of in situ vaccination and simultaneous targeting of checkpoint inhibitors responsible for T cell anergy and/or exhaustion.

Finally, targeting other immunosuppressive pathway could be a way to enhance responses to immunotherapy. Among those, one of the most dominant immune resistance mechanismsin EOC is mediated by indole-amine-2,3-dioxygenase (IDO), an enzyme that catalyzes the tryptophan degradation along the kynurenine pathway and enhances the synthesis of immunosuppressive metabolites [84]. Inhibition of this pathway is being explored in different tumors. 

The autologous infusion of enriched naturally occurring tumor reactive lymphocytes could be a way to improve immunotherapy efficacy in already infiltrated tumors. Advances in genetically engineering and new generation of TCR or CAR modified T cells could represent a real game changer in these cancers.

So far, TIL-based therapies have only been offered in small phase I/II studies in a few highly specialized centers mastering the complexity of TIL production and the very intensive nature of the three-step treatment protocol. Despite the limited data available in OC, we believe that this strategy is a unique opportunity to enhance immune-response “heating the fire” against OC.

A better characterization of the tumor and stromal environment, which shuts out TILs in immune-excluded EOCs, is critical in order to identify reliable predictive biomarkers and advance the field of immunotherapy in this malignancy. In conclusion, understanding the tumor immune escape mechanisms will enable a greater sophistication in immunotherapy treatments allowing tailoring treatment not only according to tumor biology but also to TME characteristics, extending the benefit of immunotherapy to more patients and hopefully providing long-lasting durable responses. 

## Figures and Tables

**Table 1:** Ongoing phase III clinical trials exploring CPIs and anti- vascular endothelial grow factor (VEGF).

Trial Name	NCT Code	Setting	Patients’ selection	Arms	Primary Endpoints
GOG3015/ENGOT OV39	NCT03038100	Front line	Stage III or IV PDS or IDS any residual Stratification PD-L1 0 vs 1+	Carbo-Tax + Bev Carbo-Tax + Bev + Atezo	PFS and PFS in PD-L1 + subpop OS and OS in PD-L1 + subpop
ATALANTE/ENGOT OV29	NCT02891824	Recurrence	1 or 2 previous CT lines PFI > 6 months Stratification PD-L1	Carbo combo + Bev Carbo combo + Bev + Atezo	PFS
EORTC-1508	NCT02659384	Recurrence	Platinum resistant Any num of platinum lines Max 2 lines non-platinum Biopsy required	Bev + aspirin Bev + Atezo + aspirin Bev + Atezo + placebo Atezo + aspirin Atezo + placebo	PFS at 6 months

PDS: Primary debulking surgery; IDS: Interval debulking surgery; PD-L1: Programmed death-ligand 1; CT: Chemotherapy; PFI: Platinum-free interval; num: Number; max: Maximum; Carbo: Carboplatin; Tax: Taxol; Bev: Bevacizumab; Atezo: Atezolizumab; combo: Combination; PFS: Progression-free survival; subpop: Subpopulation; OS: Overall survival.

**Table 2:** Ongoing phase III clinical trials exploring combination of CPIs and poly (ADP-ribose) polymerase (PARP) inhibitors (PARPIs).

Trial Name	NCT Code	Setting	Patients’ Selection	Arms	Endpoints
AGO/DUO-ENGOT Ov46	NCT03737643	Front line	BRCA non-mut PDS or IDS any residual LGSOC excluded	Carbo-tax+Bev+placebo+palcebo Carbo-tax+Bev+durvalumab+placebo Carbo-tax+Bev+durvalumab+olaparib	PFS in BRCA non-mut
BGOG/ENGOT Ov43	NCT03740165	Front line	BRCA non-mut any histotype PDS or IDS any residual Bev optional	Carbo-tax+placebo+placebo Carbo-tax+pembrolizumab+placebo Carbo-tax+pembrolizumab+olaparib	PFS, OS
GINECO/FIRST ENGOT Ov44	NCT03602859	Front line	PDS or IDS Mucinous excluded Bev optional	Carbo-tax+palcebo+placebo Carbo-tax+placebo+niraparib Carbo-tax+TSR042+niraparib	PFS
ATHENA GOG3020/ENGOT Ov45	NCT03522246	Maintenance after front line	Stage III-IV HGSOC PDS or IDS Response to platinum	Rucaparib-nivolumab Rucaparib-placebo Nivolumab-placebo Placebo-placebo	PFS

BRCA: Breast cancer related genes; PDS: Primary debulking surgery; IDS: Interval debulking surgery; LGSOC: Low grade serous ovarian cancer; HGSOC: High-grade serous ovarian cancer; Carbo: Carboplatin; Tax: Taxol; Bev: Bevacizumab; PFS: Progression-free survival; OS: Overall survival.

**Table 3:** Selected trials of adoptive therapies in epithelial ovarian cancer (EOC).

Therapy	NCT Code	Phase	Patients’ Selection	Primary Endpoint(s)
TILs, IL2 and pembrolizumab after Cy	NCT03158935	I	Platinum resistant	SAE within 35 days of TIL infusion
TILs and IL2 after Cy and F with prior treatment with ipilimumab and nivolumab	NCT03287674	I/II	Platinum resistant If platinum sensitive ≥ 2 previous lines	AE
Cy and TAEST16001 (NY-ESO1 specific TCR-T cell)	NCT03159585	I	Further lines HLA-A*0201 POSITIVE NY-ESO-1 positive cells≥25% by IHC	AE (30 days)
Genetically engeneered NY-ESO-1 specific lymphocycites (IV et IP), aldesleukin after Cy and decitabine	NCT03017131	I	Platinum resistant If platinum sensitive ≥2 previous lines HLA-A*0201 POSITIVE	AE (28 days)
TBI-1201 (MAGE-A4-specific TCR transduced T lymphocites) after Cy and F	NCT02096614	I	HLA-A*24:02 positive MAGE-A4-expression by PCR or IHC	/
4H11-28z/fIL-12/EGFRt+ Genetically-modified T cells after Cy	NCT02498912	I	Further lines MUC16ecto positive tumors in IHC (score 3–5)	MTD (30 days)
Anti-mesothelin CAR transduced PBL and aldesleukin after Cy and F	NCT01583686	I/II	Further lines	AE (5 years), ORR
Anti-HER2 CAR-T cells	NCT02713984	I/II	Further lines HER2+ cells	AE

TILs: Tumor infiltrating lymphocytes; IL-2: Interleukin-2; Cy: Cyclophosphamide; F: Fludarabine; IV: Intravenously; IP: Intraperitoneally; AE: Adverse events; TCR: T cell receptor; CAR-T: Chimeric antigen receptor-T cell; PBL: Peripheral blood lymphocytes.

## References

[1] Siegel R.L., Miller K.D., Jemal A. (2019). Cancer statistics, 2019. CA Cancer J. Clin..

[2] Ledermann J.A., Raja F.A., Fotopoulou C., Gonzalez-Martin A., Colombo N., Sessa C., ESMO Guidelines Working Group (2013). Newly diagnosed and relapsed epithelial ovarian carcinoma: ESMO Clinical Practice Guidelines for diagnosis, treatment and follow-up. Ann. Oncol..

[3] Mirza M.R., Pignata S., Ledermann J.A. (2018). Latest clinical evidence and further development of PARP inhibitors in ovarian cancer. Ann. Oncol..

[4] Miller K.D., Siegel R.L., Lin C.C., Mariotto A.B., Kramer J.L., Rowland J.H., Stein K.D., Alteri R., Jemal A. (2016). Cancer treatment and survivorship statistics, 2016. CA Cancer J. Clin..

[5] Schlienger K., Chu C.S., Woo E.Y., Rivers P.M., Toll A.J., Hudson B., Maus M.V., Riley J.L., Choi Y., Coukos G. (2003). TRANCE-and CD40 ligand-matured dendritic cells reveal MHC class I-restricted T cells specific for autologous tumor in late-stage ovarian cancer patients. Clin. Cancer Res..

[6] Goodell V., Salazar L.G., Urban N., Drescher C.W., Gray H., Swensen R.E., McIntosh M.W., Disis M.L. (2006). Antibody immunity to the p53 oncogenic protein is a prognostic indicator in ovarian cancer. J. Clin. Oncol..

[7] Oda K., Hamanishi J., Matsuo K., Hasegawa K. (2018). Genomics to immunotherapy of ovarian clear cell carcinoma: Unique opportunities for management. Gynecol. Oncol..

[8] Strickland K.C., Howitt B.E., Shukla S.A., Rodig S., Ritterhouse L.L., Liu J.F., Garber J.E., Chowdhury D., Wu C.J., D’Andrea A.D. (2016). Association and prognostic significance of BRCA1/2-mutation status with neoantigen load, number of tumor-infiltrating lymphocytes and expression of PD-1/PD-L1 in high grade serous ovarian cancer. Oncotarget.

[9] Wieser V., Gaugg I., Fleischer M., Shivalingaiah G., Wenzel S., Sprung S., Lax S.F., Zeimet A.G., Fiegl H., Marth C. (2018). *BRCA1/2* and *TP53* mutation status associates with *PD-1* and *PD-L1* expression in ovarian cancer. Oncotarget.

[10] Zhang L., Conejo-Garcia J.R., Katsaros D., Gimotty P.A., Massobrio M., Regnani G., Makrigiannakis A., Gray H., Schlienger K., Liebman M.N. (2003). Intratumoral T cells, recurrence, and survival in epithelial ovarian cancer. N. Engl. J. Med..

[11] Curiel T.J., Coukos G., Zou L., Alvarez X., Cheng P., Mottram P., Evdemon-Hogan M., Conejo-Garcia J.R., Zhang L., Burow M. (2004). Specific recruitment of regulatory T cells in ovarian carcinoma fosters immune privilege and predicts reduced survival. Nat. Med..

[12] Hamanishi J., Mandai M., Iwasaki M., Okazaki T., Tanaka Y., Yamaguchi K., Higuchi T., Yagi H., Takakura K., Minato N. (2007). Programmed cell death 1 ligand 1 and tumor-infiltrating CD8+ T lymphocytes are prognostic factors of human ovarian cancer. Proc. Natl. Acad. Sci. USA.

[13] Gabrilovich D.I., Nagaraj S. (2009). Myeloid-derived suppressor cells as regulators of the immune system. Nat. Rev. Immunol..

[14] Buckanovich R.J., Facciabene A., Kim S., Benencia F., Sasaroli D., Balint K., Katsaros D., O’Brien-Jenkins A., Gimotty P.A., Coukos G. (2008). Endothelin B receptor mediates the endothelial barrier to T cell homing to tumors and disables immune therapy. Nat. Med..

[15] Kandalaft L.E., Facciabene A., Buckanovich R.J., Coukos G. (2009). Endothelin B receptor, a new target in cancer immune therapy. Clin. Cancer Res..

[16] Champiat S., Ileana E., Giaccone G., Besse B., Mountzios G., Eggermont A., Soria J.C. (2014). Incorporating immune-checkpoint inhibitors into systemic therapy of NSCLC. J. Thorac. Oncol..

[17] Alme A.K., Karir B.S., Faltas B.M., Drake C.G. (2016). Blocking immune checkpoints in prostate, kidney, and urothelial cancer: An overview. Urol. Oncol..

[18] Hamanishi J., Mandai M., Ikeda T., Minami M., Kawaguchi A., Murayama T., Kanai M., Mori Y., Matsumoto S., Chikuma S. (2015). Safety and Antitumor Activity of Anti-PD-1 Antibody, Nivolumab, in Patients With Platinum-Resistant Ovarian Cancer. J. Clin. Oncol..

[19] Varga A., Piha-Paul S.A., Ott P.A., Mehnert J.M., Berton-Rigaud D., Johnson E.A., Cheng J.D., Yuan S., Rubin E.H., Matei D.E. (2015). Antitumor activity and safety of pembrolizumab in patients (pts) with PD-L1 positive advanced ovarian cancer: Interim results from a phase Ib study. J. Clin. Oncol..

[20] Disis M.L., Patel M.R., Pant S., Hamilton E.P., Lockhart A.C., Kelly K., Beck J.T., Gordon M.S., Weiss G.J., Taylor M.H. (2016). Avelumab (MSB0010718C; anti-PD-L1) in patients with recurrent/refractory ovarian cancer from the JAVELIN Solid Tumor phase Ib trial: Safety and clinical activity. J. Clin. Oncol..

[21] Infante J.R., Braiteh F., Emens L.A., Balmanoukian A.S., Oaknin A., Wang Y., Liu B., Molinero L., Fasso M., O’Hear C. (2016). Safety, clinical activity and biomarkers of atezolizumab (atezo) in advanced ovarian cancer (OC). Ann. Oncol..

[22] Burger R., Sill M., Zamarin D. NRG Oncology phase II randomized trial of nivolumab with or without ipilimumab in patients with persistent or recurrent ovarian cancer. Proceedings of the 17th Biennial Meeting of the International Gynecologic Cancer Society.

[23] Lanitis E., Dangaj D., Irving M., Coukos G. (2017). Mechanisms regulating T cell infiltration and activity in solid tumors. Ann. Oncol..

[24] Hegde P.S., Karanikas V., Evers S. (2016). The Where, the When, and the How of Immune Monitoring for Cancer Immunotherapies in the Era of Checkpoint Inhibition. Clin. Cancer Res..

[25] Galon J., Bruni D. (2019). Approaches to treat immune hot, altered and cold tumours with combination immunotherapies. Nat. Rev. Drug Discov..

[26] Fukumura D., Kloepper J., Amoozgar Z., Duda D.G., Jain R.K. (2018). Enhancing cancer immunotherapy using antiangiogenics: Opportunities and challenges. Nat. Rev. Clin. Oncol..

[27] Voron T., Colussi O., Marcheteau E., Pernot S., Nizard M., Pointet A.L., Latreche S., Bergaya S., Benhamouda N., Tanchot C. (2015). VEGF-A modulates expression of inhibitory checkpoints on CD8+ T cells in tumors. J. Exp. Med..

[28] Gabrilovich D., Ishida T., Oyama T., Ran S., Kravtsov V., Nadaf S., Carbone D.P. (1998). Vascular endothelial growth factor inhibits the development of dendritic cells and dramatically affects the differentiation of multiple hematopoietic lineages in vivo. Blood.

[29] Khan K.A., Kerbel R.S. (2018). Improving immunotherapy outcomes with anti-angiogenic treatments and vice versa. Nat. Rev. Clin. Oncol..

[30] Lee J.M., Cimino-Mathews A., Peer C.J., Zimmer A., Lipkowitz S., Annunziata C.M., Cao L., Harrell M.I., Swisher E.M., Houston N. (2017). Safety and Clinical Activity of the Programmed Death-Ligand 1 Inhibitor Durvalumab in Combination With Poly (ADP-Ribose) Polymerase Inhibitor Olaparib or Vascular Endothelial Growth Factor Receptor 1-3 Inhibitor Cediranib in Women’s Cancers: A Dose-Escalation, Phase I Study. J. Clin. Oncol..

[31] Hirte H., Lheureux S., Fleming G.F., Sugimoto A., Morgan R., Biagi J., Wang L., McGill S., Ivy S.P., Oza A.M. (2015). A phase 2 study of cediranib in recurrent or persistent ovarian, peritoneal or fallopian tube cancer: A trial of the Princess Margaret, Chicago and California Phase II Consortia. Gynecol. Oncol..

[32] Liu J.F., Herold C., Luo W., Penson R., Horowitz N., Konstantinopoulos P., Castro C., Curtis J., Matulonis U.A., Cannistra S. (2018). 937PD A phase 2 trial of combination nivolumab and bevacizumab in recurrent ovarian cancer. Ann. Oncol..

[33] (2014). Lymparza [Package Insert], Astra Zeneca. https://www.accessdata.fda.gov/drugsatfda_docs/label/2014/206162lbl.pdf.

[34] (2017). Lymparza [Package Insert], Astra Zeneca. https://www.accessdata.fda.gov/drugsatfda_docs/label/2017/208558s000lbl.pdf.

[35] (2018). Lymparza [Package Insert], Astra Zeneca. https://www.accessdata.fda.gov/drugsatfda_docs/label/2018/208558s001lbl.pdf.

[36] (2016). Rubraca [Package Insert] Clovis Oncology. https://www.accessdata.fda.gov/drugsatfda_docs/label/2016/209115s000lbl.pdf.

[37] (2018). Rubraca [Package Insert], Clovis Oncology. http://clovisoncology.com/files/rubraca-prescribing-info.pdf.

[38] Zejula [Package Insert]. Waltham, MA: Tesaro Inc. 2017. https://www.accessdata.fda.gov/drugsatfda_docs/label/2017/208447lbl.pdf.

[39] Aldinucci A., Gerlini G., Fossati S., Cipriani G., Ballerini C., Biagioli T., Pimpinelli N., Borgognoni L., Massacesi L., Moroni F. (2007). A key role for poly(ADP-ribose) polymerase-1 activity during human dendritic cell maturation. J. Immunol..

[40] Valdor R., Schreiber V., Saenz L., Martínez T., Munoz-Suano A., Dominguez-Villar M., Ramírez P., Parrilla P., Aguado E., García-Cózar F. (2008). Regulation of NFAT by poly(ADP-ribose) polymerase activity in T cells. Mol. Immunol..

[41] Stewart R.A., Pilié P.G., Yap T.A. (2018). Development of PARP and Immune-Checkpoint Inhibitor Combinations. Cancer Res..

[42] Nolan E., Savas P., Policheni A.N., Darcy P.K., Vaillant F., Mintoff C.P., Dushyanthen S., Mansour M., Pang J.M.B., Fox S.B. (2017). Combined immune checkpoint blockade as a therapeutic strategy for BRCA1-mutated breast cancer. Sci. Transl. Med..

[43] Adams S.F., Rixe O., McCance D., Lee J.H., Eberhardt S., Westgate S., Rutledge T., Muller C. (2017). Phase I study combining PARP-inhibition with immune checkpoint blockade in women with BRCA-deficient recurrent ovarian cancer. Gynecol. Oncol..

[44] Konstantinopoulos P.A., Waggoner S.E., Vidal G.A., Mita M.M., Fleming G.F., Holloway R.W., Van Le L., Sachdev J.C., Chapman-Davis E., Colon-Otero G. (2018). TOPACIO/Keynote-162: A phase 1/2 study of niraparib + pembrolizumab: Results from the platinum-resistant ovarian cancer (PROC) cohort. J. Clin. Oncol..

[45] Drew Y., De Jonge M., Hong S.H., Park Y.H., Wolfer A., Brown J., Ferguson M., Gore M.E., Alvarez R.H., Gresty C. (2018). An open-label, phase II basket study of olaparib and durvalumab (MEDIOLA): Results in germline *BRCA*-mutated (*gBRCA*m) platinum-sensitive relapsed (PSR) ovarian cancer (OC). Gynecol. Oncol..

[46] Lee J.M., Annunziata C.M., Houston N., Kohn E.C., Lipkowitz S., Minasian L., Nichols E., Trepel J., Trewhitt K., Zia F. (2018). 936PD A phase 2 study of durvalumab, a PD-L1 inhibitor and olaparib in recurrent ovarian cancer (OvCa). Ann. Oncol..

[47] Vogelstein B., Papadopoulos N., Velculescu V.E., Zhou S., Diaz L.A., Kinzler K.W. (2013). Cancer genome landscapes. Science.

[48] Kanchi K.L., Johnson K.J., Lu C., McLellan M.D., Leiserson M.D., Wendl M.C., Zhang Q., Koboldt D.C., Xie M., Kandoth C. (2014). Integrated analysis of germline and somatic variants in ovarian cancer. Nat. Commun..

[49] Deniger D.C., Pasetto A., Robbins P.F., Gartner J.J., Prickett T.D., Paria B.C., Malekzadeh P., Jia L., Yossef R., Langhan M.M. (2018). T-cell Responses to *TP53* “Hotspot” Mutations and Unique Neoantigens Expressed by Human Ovarian Cancers. Clin. Cancer Res..

[50] Bobisse S., Genolet R., Roberti A., Tanyi J.L., Racle J., Stevenson B.J., Iseli C., Michel A., Bitoux M.A., Guillaume P. (2018). Sensitive and frequent identification of high avidity neo-epitope specific CD8 + T cells in immunotherapy-naive ovarian cancer. Nat. Commun..

[51] Westergaard M.C.W., Andersen R., Chong C., Kjeldsen J.W., Pedersen M., Friese C., Hasselager T., Lajer H., Coukos G., Bassani-Sternberg M. (2019). Tumour-reactive T cell subsets in the microenvironment of ovarian cancer. Br. J. Cancer.

[52] Fujita K., Ikarashi H., Takakuwa K., Kodama S., Tokunaga A., Takahashi T., Tanaka K. (1995). Prolonged disease-free period in patients with advanced epithelial ovarian cancer after adoptive transfer of tumor-infiltrating lymphocytes. Clin. Cancer Res..

[53] Pedersen M., Westergaard M.C.W., Milne K., Nielsen M., Borch T.H., Poulsen L.G., Hendel H.W., Kennedy M., Briggs G., Ledoux S. (2018). Adoptive cell therapy with tumor-infiltrating lymphocytes in patients with metastatic ovarian cancer: A pilot study. Oncoimmunology.

[54] Pölcher M., Rudlowski C., Friedrichs N., Mielich M., Höller T., Wolfgarten M., Kübler K., Büttner R., Kuhn W., Braun M. (2010). In vivo intratumor angiogenic treatment effects during taxane-based neoadjuvant chemotherapy of ovarian cancer. BMC Cancer.

[55] Peng J., Hamanishi J., Matsumura N., Abiko K., Murat K., Baba T., Yamaguchi K., Horikawa N., Hosoe Y., Murphy S.K. (2015). Chemotherapy induces programmed cell death-ligand 1 overexpression via the nuclear factor-kappaB to foster an immunosuppressive tumor microenvironment in ovarian cancer. Cancer Res..

[56] Böhm S., Montfort A., Pearce O.M., Topping J., Chakravarty P., Everitt G.L., Clear A., McDermott J.R., Ennis D., Dowe T. (2016). Neoadjuvant chemotherapy modulates the immune microenvironment in metastases of tubo-ovarian high-grade serous carcinoma. Clin. Cancer Res..

[57] Lo C.S., Sanii S., Kroeger D.R., Milne K., Talhouk A., Chiu D.S., Rahimi K., Shaw P.A., Clarke B.A., Nelson B.H. (2017). Neoadjuvant Chemotherapy of Ovarian Cancer Results in Three Patterns of Tumor-Infiltrating Lymphocyte Response with Distinct Implications for Immunotherapy. Clin. Cancer Res..

[58] Kershaw M.H., Westwood J.A., Parker L.L., Wang G., Eshhar Z., Mavroukakis S.A., White D.E., Wunderlich J.R., Canevari S., Rogers-Freezer L. (2006). A phase I study on adoptive immunotherapy using gene-modified T cells for ovarian cancer. Clin. Cancer Res..

[59] Song D.G., Powell D.J. (2012). Pro-survival signaling via CD27 costimulation drives effective CAR T-cell therapy. Oncoimmunology.

[60] Canevari S., Stoter G., Arienti F., Bolis G., Colnaghi M.I., Di Re E.M., Eggermont A.M., Goey S.H., Gratama J.W., Lamers C.H. (1995). Regression of advanced ovarian carcinoma by intraperitoneal treatment with autologous T lymphocytes retargeted by a bispecific monoclonal antibody. J. Natl. Cancer Inst..

[61] Hassan R., Thomas A., Alewine C., Le D.T., Jaffee E.M., Pastan I. (2016). Mesothelin Immunotherapy for Cancer: Ready for Prime Time?. J. Clin. Oncol..

[62] Tanyi J.L., Haas A.R., Beatty G.L., Stashwick C.J., O’Hara M.H., Morgan M.A., Porter D.L., Melenhorst J.J., Plesa G., Lacey S.F. (2016). Anti-mesothelin chimeric antigen receptor T cells in patients with epithelial ovarian cancer. JCO.

[63] Geller M.A., Cooley S., Judson P.L., Ghebre R., Carson L.F., Argenta P.A., Jonson A.L., Panoskaltsis-Mortari A., Curtsinger J., McKenna D. (2011). A phase II study of allogeneic natural killer cell therapy to treat patients with recurrent ovarian and breast cancer. Cytotherapy.

[64] Yang Y., Lim O., Kim T.M., Ahn Y.O., Choi H., Chung H., Min B., Her J.H., Cho S.Y., Keam B. (2016). Phase I Study of Random Healthy Donor-Derived Allogeneic Natural Killer Cell Therapy in Patients with Malignant Lymphoma or Advanced Solid Tumors. Cancer Immunol. Res..

[65] Schmeel L.C., Schmeel F.C., Coch C., Schmidt-Wolf I.G. (2015). Cytokine-induced killer (CIK) cells in cancer immunotherapy: Report of the international registry on CIK cells (IRCC). J. Cancer Res. Clin. Oncol..

[66] Kim H.M., Kang J.S., Lim J., Park S.K., Lee K., Yoon Y.D., Lee C.W., Lee K.H., Han G., Yang K.H. (2007). Inhibition of human ovarian tumor growth by cytokine-induced killer cells. Arch. Pharm. Res..

[67] Urba W.J., Clark J.W., Steis R.G., Bookman M.A., Smith J.W., Beckner S., Maluish A.E., Rossio J.L., Rager H., Ortaldo J.R. (1989). Intraperitoneal lymphokine-activated killer cell/interleukin-2 therapy in patients with intra-abdominal cancer: Immunologic considerations. J. Natl. Cancer Inst..

[68] Steis R.G., Urba W.J., VanderMolen L.A., Bookman M.A., Smith 2nd J.W., Clark J.W., Miller R.L., Crum E.D., Beckner S.K., McKnight J.E. (1990). Intraperitoneal lymphokine-activated killer-cell and interleukin-2 therapy for malignancies limited to the peritoneal cavity. J. Clin. Oncol..

[69] Stewart J.A., Belinson J.L., Moore A.L., Dorighi J.A., Grant B.W., Haugh L.D., Roberts J.D., Albertini R.J., Branda R.F. (1990). Phase I trial of intraperitoneal recombinant interleukin-2/lymphokine-activated killer cells in patients with ovarian cancer. Cancer Res..

[70] Zhou Y., Chen C.L., Jiang S.W., Feng Y., Yuan L., Chen P., Zhang L., Huang S., Li J., Xia J.C. (2018). Retrospective analysis of the efficacy of adjuvant CIK cell therapy in epithelial ovarian cancer patients who received postoperative chemotherapy. Oncoimmunology.

[71] Hamanishi J., Mandai M., Konishi I. (2016). Immune checkpoint inhibition in ovarian cancer. Int. Immunol..

[72] Drakes M.L., Mehrotra S., Aldulescu M., Potkul R.K., Liu Y., Grisoli A., Joyce C., O’Brien T.E., Stack M.S., Stiff P.J. (2018). Stratification of ovarian tumor pathology by expression of programmed cell death-1 (PD-1) and PD-ligand- 1 (PD-L1) in ovarian cancer. J. Ovarian Res..

[73] Abiko K., Matsumura N., Hamanishi J., Horikawa N., Murakami R., Yamaguchi K., Yoshioka Y., Baba T., Konishi I., Mandai M. (2015). IFN-γ from lymphocytes induces PD-L1 expression and promotes progression of ovariancancer. Br. J. Cancer.

[74] Iwai Y., Hamanishi J., Chamoto K., Honjo T. (2017). Cancer immunotherapies targeting the PD-1 signaling pathway. J. Biomed. Sci..

[75] Darb-Esfahani S., Kunze C.A., Kulbe H., Sehouli J., Wienert S., Lindner J., Budczies J., Bockmayr M., Dietel M., Denkert C. (2016). Prognostic impact of programmed cell death-1 (PD-1) and PD-ligand 1 (PD-L1) expression in cancer cells and tumor-infiltrating lymphocytes in ovarian high grade serous carcinoma. Oncotarget.

[76] Webb J.R., Milne K., Kroeger D.R., Nelson B.H. (2016). PD-L1 expression is associated with tumor-infiltrating T cells and favorable prognosis in high-grade serous ovarian cancer. Gynecol. Oncol..

[77] Mittica G., Genta S., Aglietta M., Valabrega G. (2016). Immune Checkpoint Inhibitors: A New Opportunity in the Treatment of Ovarian Cancer?. Int. J. Mol. Sci..

[78] DeLeeuw R.J., Kroeger D.R., Kost S.E., Chang P.P., Webb J.R., Nelson B.H. (2015). CD25 identifies a subset of CD4⁺FoxP3⁻ TIL that are exhausted yet prognostically favorable in human ovarian cancer. Cancer Immunol. Res..

[79] Nielsen J.S., Sahota R.A., Milne K., Kost S.E., Nesslinger N.J., Watson P.H., Nelson B.H. (2012). CD20+ tumor-infiltrating lymphocytes have an atypical CD27- memory phenotype and together with CD8+ T cells promote favorable prognosis in ovarian cancer. Clin. Cancer Res..

[80] Kroeger D.R., Milne K., Nelson B.H. (2016). Tumor-Infiltrating Plasma Cells Are Associated with Tertiary Lymphoid Structures, Cytolytic T-Cell Responses, and Superior Prognosis in Ovarian Cancer. Clin. Cancer Res..

[81] Zhang A.W., McPherson A., Milne K., Kroeger D.R., Hamilton P.T., Miranda A., Funnell T., Little N., de Souza C.P., Laan S. (2018). Interfaces of Malignant and Immunologic Clonal Dynamics in Ovarian Cancer. Cell.

[82] Matulonis U.A., Shapira-Frommer R., Santin A., Lisyanskaya A.S., Pignata S., Vergote I., Raspagliesi F., Sonke G.S., Birrer M., Provencher D.M. (2019). Antitumor Activity and Safety of Pembrolizumab in Patients with Advanced RecurrentOvarian Cancer: Results from the Phase 2 KEYNOTE-100 Study. Ann. Oncol..

[83] Cristescu R., Mogg R., Ayers M., Albright A., Murphy E., Yearley J., Sher X., Liu X.Q., Lu H., Nebozhyn M. (2018). Pan-tumor genomic biomarkers for PD-1 checkpoint blockade-based immunotherapy. Science.

[84] Odunsi K. (2017). Immunotherapy in ovarian cancer. Ann. Oncol..

[85] Hanlon D.J., Aldo P.B., Devine L., Alvero A.B., Engberg A.K., Edelson R., Mor G. (2011). Enhanced stimulation of anti-ovarian cancer CD8(+) T cells by dendritic cells loaded with nanoparticle encapsulated tumor antigen. Am. J. Reprod. Immunol..

[86] Odunsi K., Matsuzaki J., James S.R., Mhawech-Fauceglia P., Tsuji T., Miller A., Zhang W., Akers S.N., Griffiths E.A., Miliotto A. (2014). Epigenetic potentiation of NY-ESO-1 vaccine therapy in human ovarian cancer. Cancer Immunol. Res..

[87] Rodriguez-Garcia A. (2017). Minutolo NG T-cell target antigens across major gynecologic cancers. Gynecol. Oncol..

